# Phenomenology and comorbidity in late onset bipolar disorder : A comparative study

**DOI:** 10.1192/j.eurpsy.2022.421

**Published:** 2022-09-01

**Authors:** D. Mohapatra

**Affiliations:** All India Institute of Medical Sciences, Psychiatry, Bhubaneswar, India

**Keywords:** Late Onset Bipolar disorder, Phenomenology, Comorbidity

## Abstract

**Introduction:**

Bipolar disorder in later life is a complex & confounding neuropsychiatric syndrome with diagnostic & therapeutic challenges.

**Objectives:**

To assess the clinical characteristics of late onset bipolar disorder and to compare with adult onset bipolar disorder and to compare the medical co morbidity between age, sex matched healthy control group.

**Methods:**

It was a hospital based, observational, analytical and cross-sectional study conducted over 2 and half years. The patients > 60 years presenting with manic features after satisfying the inclusion and exclusion criteria were the study group. Control group -1 was selected from adult onset bipolar disorder. YMRS, MMSE, SCID were applied for both. Control group -2 was selected from age, sex, education matched normal population and the three groups were compared for co morbidity. Secondary mania cases are excluded from the study.

**Results:**

Mean age at onset was 67.4 years. 63.3% of our patients were female. H/O psychiatric illness in family is more in control group (53.3%) than in study group (26.7%) (p=0.035). 86.6% patients present with irritability. 73.3% patients were presented with aggression. Control group –irritability=90%, aggression=75% The difference is not statistically significant. Scoring of each symptom showed significant difference. It means presence & severity of delusion is significantly more in young bipolar control group.(p=0.035 for % score, p=0.015 for mean SAPS score). 70% presented with co morbidities.

**Conclusions:**

Geriatric mania shows mixed presentation compared to early onset disease. Psychotic features are more common. Late onset mania is less associated with family history. Common co morbidities are DM, HTN, Hypothyroidism, neurologic disorders.

**Disclosure:**

No significant relationships.

